# Sex-Specific Patterns of Diaphragm Phospholipid Content and Remodeling during Aging and in a Model of SELENON-Related Myopathy

**DOI:** 10.3390/biomedicines11020234

**Published:** 2023-01-17

**Authors:** Rezlène Bargui, Audrey Solgadi, Florent Dumont, Bastien Prost, Nathalie Vadrot, Anne Filipe, Andrew T. V. Ho, Ana Ferreiro, Maryline Moulin

**Affiliations:** 1 Basic and Translational Myology Laboratory, Université Paris Cité, BFA, CNRS UMR8251, F-75013 Paris, France; 2 UMS-IPSIT-SAMM, Université Paris-Saclay, INSERM, CNRS, Ingénierie et Plateformes au Service de l’Innovation Thérapeutique, F-91400 Orsay, France; 3 UMS-IPSIT-Bioinfo, Université Paris-Saclay, INSERM, CNRS, Ingénierie et Plateformes au Service de l’Innovation Thérapeutique, F-91400 Orsay, France; 4 AP-HP, Reference Centre for Neuromuscular Disorders, Institut of Myology, Neuromyology Department, Pitié-Salpêtrière Hospital, F-75013 Paris, France

**Keywords:** phospholipid, sexual dimorphism, aging, sphingomyelin, cardiolipin, mass spectrometry, myopathy, selenoprotein N

## Abstract

Growing evidence shows that the lipid bilayer is a key site for membrane interactions and signal transduction. Surprisingly, phospholipids have not been widely studied in skeletal muscles, although mutations in genes involved in their biosynthesis have been associated with muscular diseases. Using mass spectrometry, we performed a phospholipidomic profiling in the diaphragm of male and female, young and aged, wild type and *SelenoN* knock-out mice, the murine model of an early-onset inherited myopathy with severe diaphragmatic dysfunction. We identified 191 phospholipid (PL) species and revealed an important sexual dimorphism in PLs in the diaphragm, with almost 60% of them being significantly different between male and female animals. In addition, 40% of phospholipids presented significant age-related differences. Interestingly, SELENON protein absence was responsible for remodeling of 10% PL content, completely different in males and in females. Expression of genes encoding enzymes involved in PL remodeling was higher in males compared to females. These results establish the diaphragm PL map and highlight an important PL remodeling pattern depending on sex, aging and partly on genotype. These differences in PL profile may contribute to the identification of biomarkers associated with muscular diseases and muscle aging.

## 1. Introduction

Lipids are one of the four major types of biological macromolecules along with carbohydrates, proteins and nucleic acids. Of all the different lipids, phospholipids characteristically delimit cell compartments, making them indispensable to the appearance of life [1]. Phospholipids are not only essential as a biological and physical barrier, but the lipid bilayer is also a site for protein-protein interaction and signal transduction [2]. The tremendous diversity of phospholipids and their modifications are difficult to study, and this may explain why research predominantly focuses on the role of lipid classes rather than molecular entities (more than 40,000 species) [1,3,4]). With the development of mass spectrometry, identification of lipids in a biological context has been made possible since the early 90s. Mass spectrometry remains as the gold standard technique used for lipid profiling to date [5].

The importance of PLs in muscle function and dysfunction is highlighted by mutations of genes encoding enzymes involved in PL synthesis or remodeling responsible for muscular disorders (Orphanet ORPHA:352312 [4,6]). For example, mutations in the Tafazzin (*TAZ*) gene, encoding a phospholipid transacylase crucial for cardiolipin remodeling, are responsible for Barth syndrome, a devastating neuromuscular and metabolic disease characterized by skeletal myopathy and dilated cardiomyopathy [7].

Skeletal muscle function is critical for life quality and longevity. Decrease of skeletal muscle mass and the onset of muscle strength decline begin in the fourth decade of life in humans [8,9] and age-related loss of muscle mass (sarcopenia) is associated with significant morbidity and mortality [10]. Furthermore, dysfunction of the diaphragm muscle can be a direct cause of death. Aging is associated with reduced diaphragm force and fatigue [11,12] and exhibits gender bias [13]. Diaphragmatic dysfunction and life-threatening respiratory insufficiency are also characteristic elements of various inherited and acquired myopathies [14]. Human *SELENON* gene mutations cause *SELENON(SEPN1)*-related congenital myopathy, characteristically associated with diaphragmatic dysfunction from childhood that is lethal in the absence of ventilator support [15,16,17].

Sexual dimorphism in age-related changes in skeletal muscle metabolism has been reported [18], but the mechanisms are far from being elucidated. As many investigations are still conducted in single-sex studies, limited information is available for sex-dependence in muscle PL composition. The objective of this study was to establish the PL map in diaphragm, their evolution with aging and the impact of sex or genetic disease leading to diaphragm dysfunction. We used ultra-high-resolution mass spectrometry to carry out quantitative and qualitative PL analysis in diaphragms from young (1 month) and aged (20 months), wild type and *SelenoN* KO, male and female mice. We show that sex is a major influencing factor in diaphragm PL content, reveal its changes with aging and, to a lesser extent, with SELENON protein absence.

## 2. Materials and Methods

Animals

The *SelenoN* KO mice have been described previously [19]. One-month- and 20-month-old male and female mice housed in standard animal facility conditions were culled following cervical dislocation. Diaphragms were rapidly excised and flash-frozen in liquid nitrogen for further biochemical determinations. Animal experimental procedures followed the French and European directive 2010/63/EU and regulations on the care and use of laboratory animals.

Lipid Extraction and phospholipidomic analyses by liquid chromatography coupled with Mass Spectrometry

Total lipid extraction has been described previously [20]. Briefly, the Folch method was used to extract the lipids from 5 to 15 mg of diaphragm homogenized in phosphate-buffered saline (PBS). Protein quantification was performed by BCA. A volume of total extracted lipid equivalent to 100 µg of protein was injected into a PVA-Sil column (150 × 2.1 mm I.D., 120 A) (YMC Co., Kyoto, Japan). For the phospholipid analysis, total lipid was subjected to high-performance liquid chromatography (UltiMate 3000 RSLC system—Thermo Fisher Scientific, Germering, Germany) coupled to a mass spectrometer (LC-MS) combining rapid LTQ ion trap data acquisition with an Orbitrap Velos Pro for the lipidomic study (Thermo Fisher Scientific, Bremen, Germany) and detailed in [20]. For all analyses, random sampling was used.

Real-time quantitative RT-PCR analysis

Frozen diaphragm samples were weighed and homogenized (Bertin Precellys 24) in trizol reagent (Invitrogen). RNA extraction was performed with the Direct-zol RNA kit according to manufacturer procedure (Zymo research). cDNAs were synthesized from 0.1 µg total RNA according to the protocol provided with the SuperScript III first strand synthesis system (Invitrogen). Quantitative real-time PCR was performed in duplicate using SYBR Green I master mix (Roche) with a LightCycler 480 II (Roche). Nine housekeeping genes were analyzed, and relative gene expression was analyzed using RPL19 and HPRT. Table 1 listed the primers used for each gene amplification.

Statistical analyses

All data analysis was performed using R [21] and Graphpad Prism 6. After conversion from raw files to mzXML files with Proteowizard MS Convert (Version: 3.0.19194-9338c77b2), MS data were submitted to chromatogram builder, alignment and gap filling using xcms package v3.8.0 [22]. We get a raw data matrix of 954 mass ions as variables for 33 samples distributed as n = 4 for each group, except young male WT and KO with n = 5 and aged male KO with n = 3. Zero values were replaced by matrix row medians, and data were log 2 transformed as a normalization step for downstream analysis. Then we applied a procedure based on retention time, *m*/*z* range and even or odd *m*/*z* to extract phospholipid ions. We found 268 ions that we used for supervised analysis. To find ions with differential abundance we applied a three-way analysis of variance including AGE, GENOTYPE and SEX factor. We also include combination interactions between AGE, GENOTYPE, SEX and made Tukey’s post hoc tests between pairwise groups. *p*-values (from a two-tailed test) are presented to at least three decimal places if significant and two if not. Statistical significance was defined as * for *p* < 0.05, ** for *p* < 0.01, *** for *p* < 0.001, **** for *p* < 0.0001 and ns for not significant (*p* > 0.05).

## 3. Results

### 3.1. Anatomical Parameters Influenced by Age, Sex and Genotype

**Figure 1:**
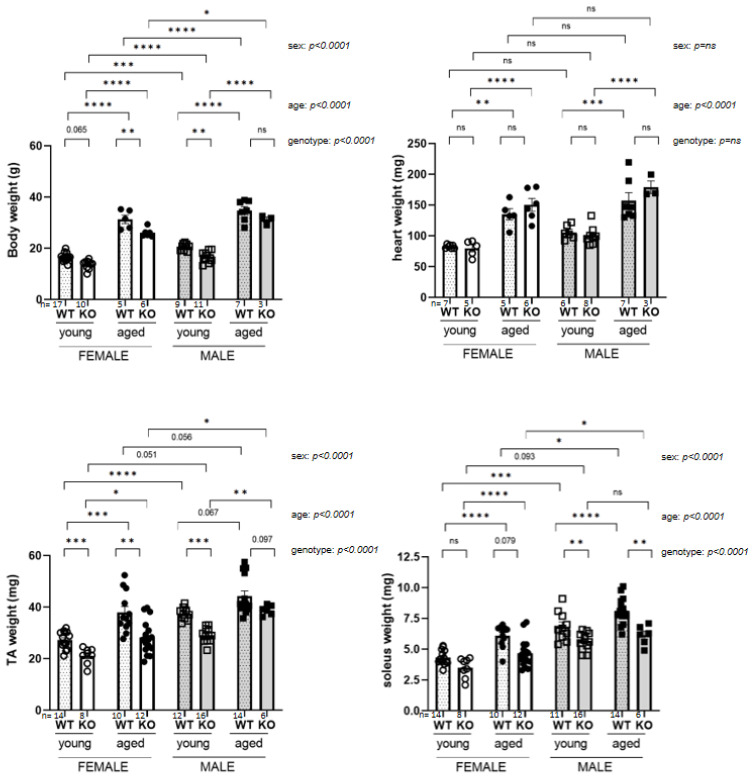
Anatomical parameters of WT and *SelenoN* KO 1-month (young) and 20-month-old (aged) male and female mice (TA for tibialis anterior). In the graph, *p*-value are from Tukey’s post hoc tests between pairwise groups. Next to the graph, *p*-values for sex, age and SelenoN effect are defined in Table 2. * *p* < 0.05, ** *p* < 0.01, *** *p* < 0.001, **** *p* < 0.0001 and ns not significant.

Significant differences in body weight (bw), heart weight, tibialis anterior (TA) and soleus muscle weight were apparent in young (1 month old) versus aged mice (20 months old). Sex and genotype also induced differences in bw, TA and soleus mass, heart weight being unaffected (cf. Figure 1 and subfigures). *SelenoN* KO mice showed lower bw (which reached significance only for aged females and young males), lower TA weight (significant for all except aged males) and reduced soleus weight (significantly only for males independently of their age).

### 3.2. Phospholipidomic Analysis Revealed an Important Sex Difference in Diaphragm PL Content

**Figure 2:**
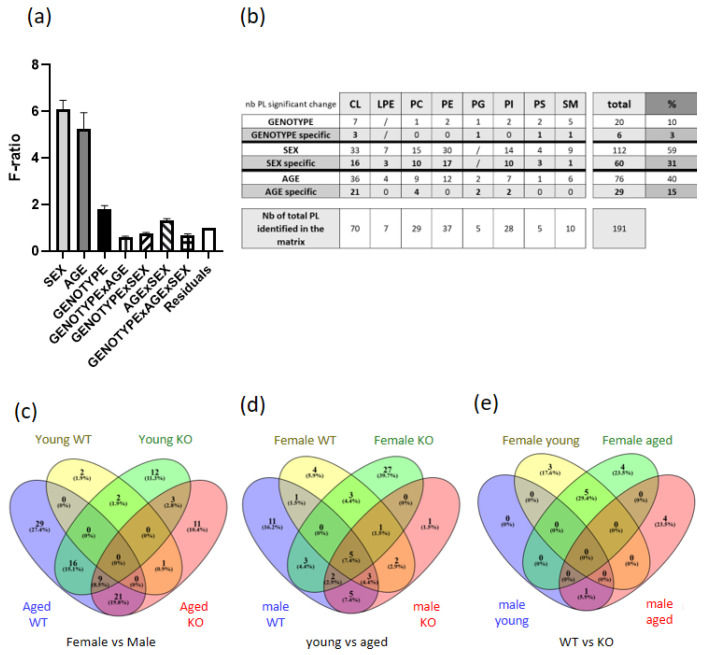
Diaphragm phospholipidomic investigation. (**a**) F-ratio analysis. (**b**) ANOVA analysis. (**c**) Number of significant PLs between male and female in young WT, young KO, aged WT and aged KO groups. (**d**) Number of significantly different PLs between young and aged in female WT, female KO, male WT and male KO groups. (**e**) Number of significantly different PLs between WT and KO in female young, female aged, male young and male aged groups.

To evaluate the diaphragm phospholipid content, we used an unbiased approach based on ultra-high resolution mass spectrometry to carry out semi-quantitative and qualitative PL analyses. We extracted 268 PLs in the diaphragm, on which we performed an analysis of variance F-ratio (cf. Figure 2a). Unexpectedly, sex, age, and to a lesser extent genotype (*SelenoN* KO) best explained the variance. ANOVA was then performed and followed by post hoc pairwise analysis to obtain *p*-values for each comparison for each phospholipid. We carried out a detailed identification and obtained 191 non-redundant PLs (cf. Figure 2b). From the 191 identified PLs, 60 were significantly different only between males and females (sex-specific effect). However, taking into account the difference in sex as well as the effects of age and genotype, 112 PLs were significantly different (cf. Figure 2b and Table 2). These results showed that 31% of PL species were influenced specifically by sex, 15% by age and 3% by the absence of SELENON (genotype). Using a Venn diagram, we observed that phospholipids that were significantly different between males and females (i.e., sex differences) were relatively distinct for each group: young WT (two specific PLs), young KO (12 specific PLs), aged WT (29 specific PLs) and aged KO mice (11 specific PLs) (cf. Figure 2c). There were significantly different PLs between young and aged which were not equal in female WT (four specific PLs), female KO (27 specific PLs), male WT (11 specific PLs) and male KO groups (one unique PLs) (cf. Figure 2d). However, we observed that the same five PLs significantly differed between young and aged in all sexes and genotypes. These PLs associated with age in all four groups (female WT, female KO, male WT and male KO) all belonged to the cardiolipin (CL) family containing 74 carbons (three chains of 18 carbons and one of 20) with seven or eight unsaturations and one to four oxidations (CL74:7, CL 74:7 +2O, CL74:8 +O, CL74:8 +2O, CL74:8 +4O). The significantly different PLs between wild-type or KO context were specific to each group, except for five PLs deregulated both in young and aged KO females (cf. Figure 2e). These five PLs were also cardiolipins but contained short and medium fatty acyl chains, mainly saturated or monounsaturated with global identification of 68:1/68:2/68:3 and 70:3 and 70:4.

### 3.3. Sex-Specific PL Remodeling in More Than Half of PLs

**Figure 3:**
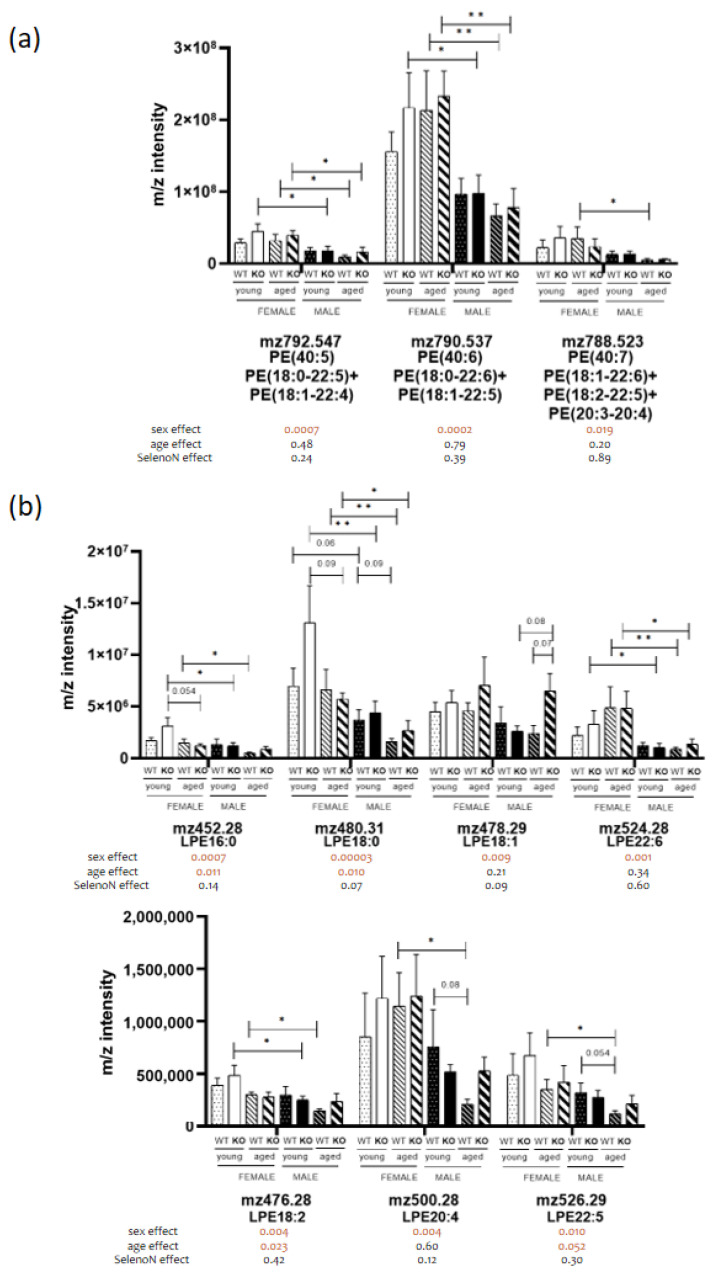
Ethanolamine-containing PLs in diaphragm. (**a**) Analysis of *m*/*z* intensity of PE species (mean ± SEM; n = 4 for each group, except young male WT and KO with n = 5 and aged male KO with n = 3). (**b**) Analysis of *m*/*z* intensity of LPE species. In the graph, *p*-value are from Tukey’s post hoc tests between pairwise groups. Under the graph, *p*-values for sex, age and SelenoN effect are defined in Table 2. * *p* < 0.05 and ** *p* < 0.01.

**Figure 4:**
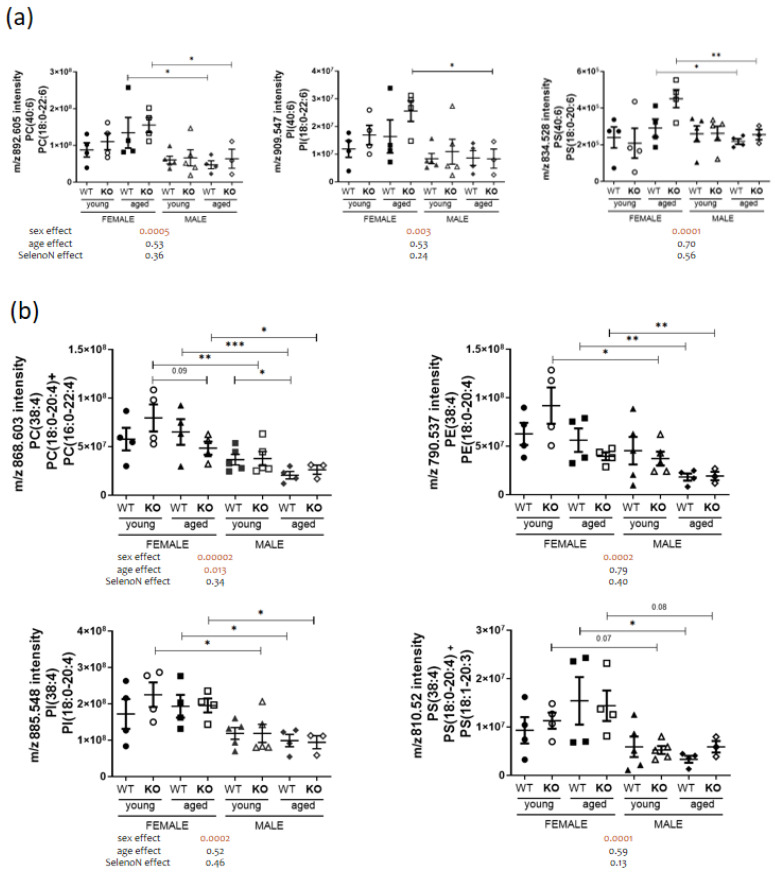
PL containing long fatty acyl chains in diaphragm present sex dimorphism. (**a**) Analysis of *m*/*z* intensity of PL (40:6) species (mean ± SEM; n = 4 for each group, except young male WT and KO with n = 5 and aged male KO with n = 3). (**b**) Analysis of *m*/*z* intensity of PL (38:4) species. In the graph, *p*-values are from Tukey’s post hoc tests between pairwise groups. Under the graph, *p*-values for sex, age and SelenoN effect are defined in Table 2. * *p* < 0.05, ** *p* < 0.01 and *** *p* < 0.001.

Global phospholipid diaphragm content was largely different between males and females (cf. Figure 2b). The most noticeable specific sex dimorphism was found in the phosphatidylethanolamine (PE) family, which is the second most abundant PL in eukaryotes (cf. Figure 2a). Despite the large number of PE identified, none were specifically different in terms of genotype and age (cf. Figure 2b). We observed an importantly reduced content of PE containing long acyl chains (≥20 carbons) such as docosapentaenoic (22:5) and docosahexaenoic (22:6) acyl chains only in males (cf. Figure 3a). Interestingly, the same profile was present for the lysophosphatidylethanolamine (cf. Figure 3b). Sex differences were also observed in other PLs containing 22:6 acyl chains (40:6), such as phosphatidylcholine (PC), phosphatidylinositol (PI) and phosphatidylserine (PS) (cf. Figure 4a) and in PLs with 38 carbons and four unsaturations mainly composed of 18:0 and 20:4 acyl chains (cf. Figure 4b). Noticeably, we were not able to detect cardiolipins containing 22:5 and 22:6 acyl chains. These data are to some extend in accordance with our previous results showing sex differences in heart muscle, males having a lower content of PLs with longer acyl chains [23].

### 3.4. Sphingomyelin and Cardiolipin Changes in Diaphragm

**Figure 5:**
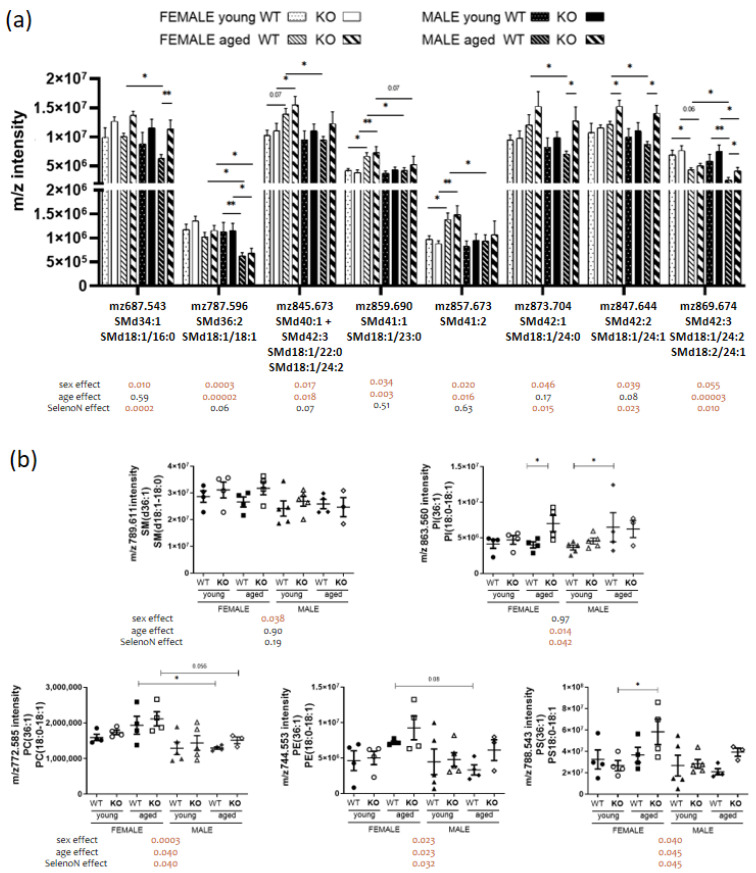
Sphingomyelin and PL (36:1) remodeling in a sex, age and/or *SelenoN* KO context. (**a**) Analysis of *m*/*z* intensity of SM species (mean ± SEM; n = 4 for each group, except young male WT and KO with n = 5 and aged male KO with n = 3). (**b**) Analysis of *m*/*z* intensity of PL (36:1) species. In the graph, *p*-value are from Tukey’s post hoc tests between pairwise groups. Under the graph, *p*-values for sex, age and SelenoN effect are defined in Table 2. * *p* < 0.05 and ** *p* < 0.01.

Next, we analyzed two particular PL classes, sphingomyelin (SM), which is composed of a sphingosine and a fatty acid, and cardiolipin, which is a PL containing four acyl chains and present only in mitochondria membranes. We observed that sphingomyelin remodeling depends either on sex, age and/or SELELON absence (cf. Figure 2b and Figure 5a). Nine out of 10 identified SM presented significant sex differences. However, SM content remodeling was also associated with a change due to age or genotype or both. The only SM which was specifically different for sex was SM (d36:1) with a decreased content in male (cf Figure 5b). Of note, this PL (36:1) content, corresponding to 18:0 and 18:1 acyl chains, was modulated by the three parameters for PC, PE and PS, and only for age and SELENON absence for PI.

**Figure 6:**
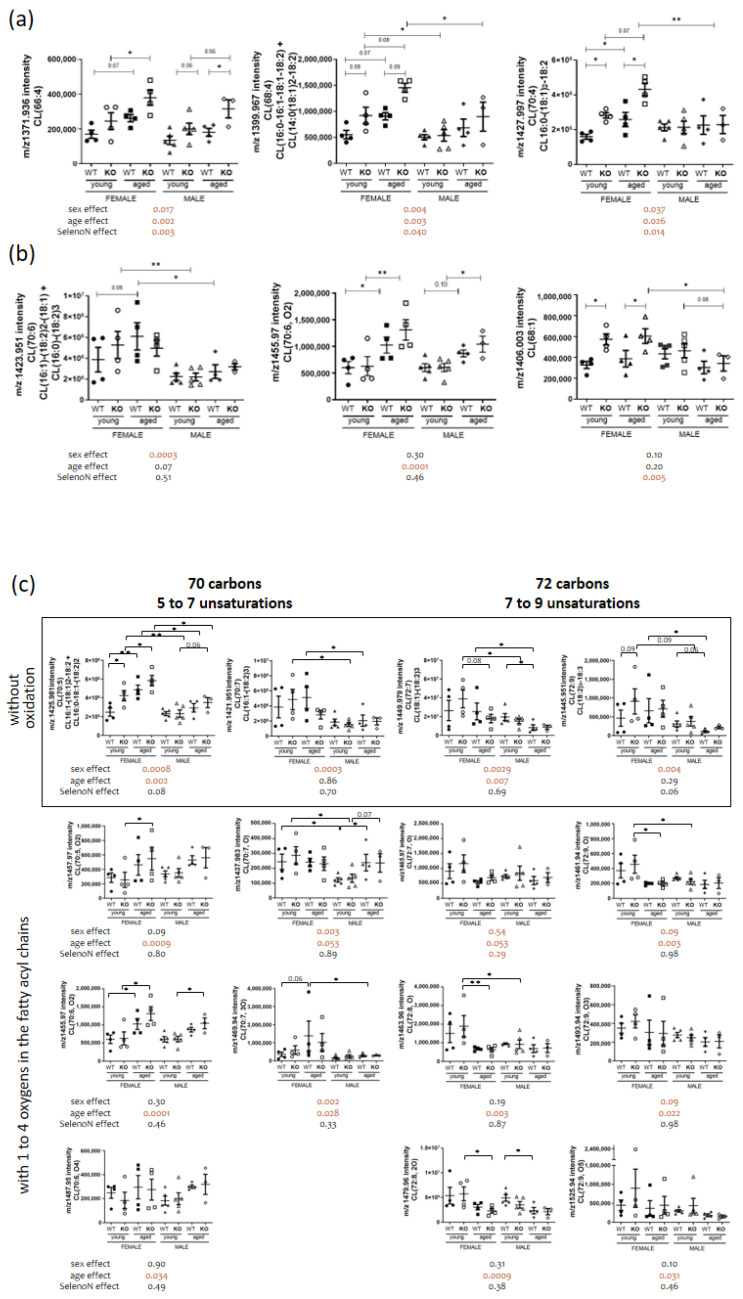

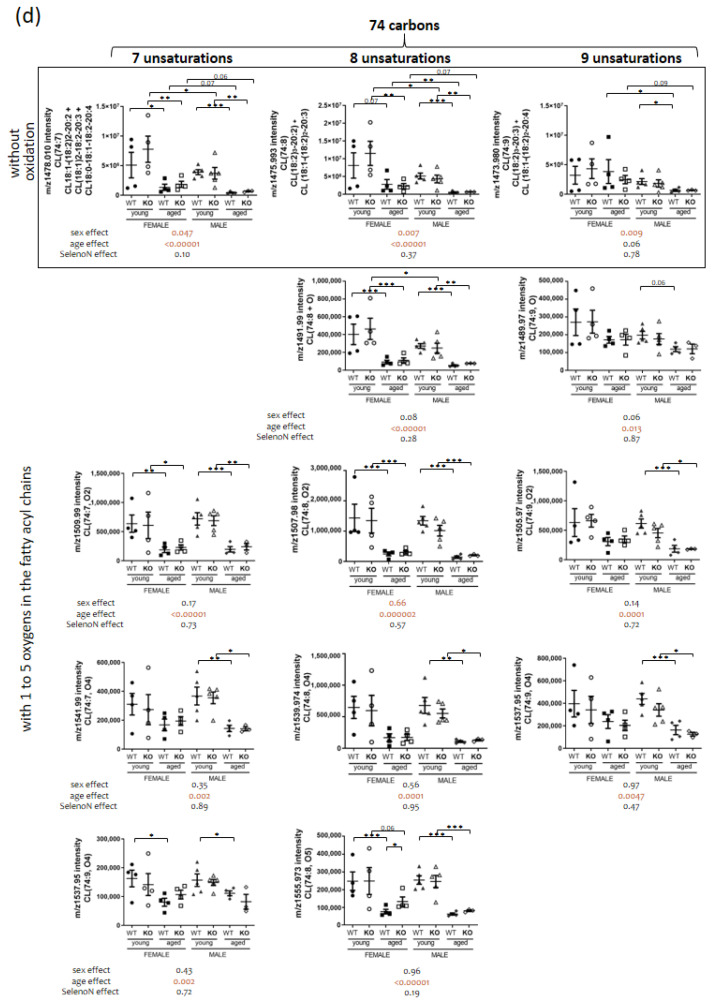
Cardiolipin remodeling in a sex, age and/or *SelenoN* KO context. (**a**) Analysis of *m*/*z* intensity of CL species with combined sex, age and *SelenoN* effects (mean ± SEM; n = 4 for each group, except young male WT and KO with n = 5 and aged male KO with n = 3). (**b**) Analysis of *m*/*z* intensity of CL species with sex, age or *SelenoN* significant differences. (**c**) Analysis of *m*/*z* intensity of CL containing 70 or 72 carbons. (**d**) Analysis of *m*/*z* intensity of CL containing 74 carbons. In the graph, *p*-value are from Tukey’s post hoc tests between pairwise groups. Under the graph, *p*-values for sex, age and SelenoN effect are defined in Table 2. * *p* < 0.05, ** *p* < 0.01 and *** *p* < 0.001.

As with sphingomyelin, many changes in diaphragm cardiolipin content were observed for the 70 species identified. Interestingly, only three CL were modulated by sex, age and genotype (cf. Figure 6a), namely cardiolipins containing four unsaturated bonds at 66, 68 or 70 carbons. We observed sex-specific effects on CL containing at least five unsaturated bonds, with decreased content in males (cf. Figure 6b first panel and Table 2). Importantly, more than half of cardiolipins were altered with aging (cf. Figure 2b and Table 3). Also, specific effects on CL containing oxidation with increased content for aged animals (cf. Figure 6b middle panel). Only 10% of cardiolipins were modified in the absence of SELENON with a specific effect on CL containing 68 carbons with one to three unsaturations with increased content in *SelenoN* KO muscles (cf. Figure 6b last panel and Table 4).

### 3.5. Modifications in PL and Fatty Acid Biosynthesis Pathways

**Figure 7:**
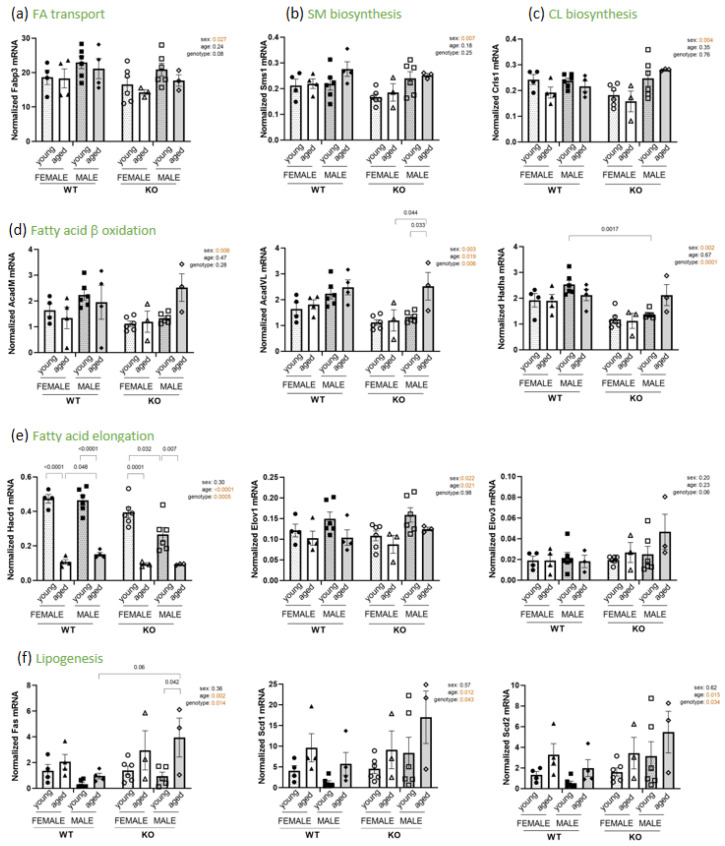
Lipid biosynthesis, remodeling and transport pathways. mRNA expression analysis in male and female, young and aged and WT and *SelenoN* KO diaphragm normalized to RPL19 and HPRT mean. (**a**) *Fabp3* (fatty acid binding protein3) involved in FA transport. (**b**) *Sms1* (sphingomyelin synthase 1) involved in SM biosynthesis. (**c**) *Crls1* (cardiolipin synthase1) involved in CL biosynthesis. (**d**) *AcadM* (acyl-coA dehydrogenase medium chain), *AcadVL* (acyl-coA dehydrogenase very long chain) and *Hadha* (hydroxyacyl-coA dehydrogenase alpha) involved in FA beta oxidation. (**e**) *Hacd1* (3-hydroxyacyl-coA dehydratase 1), *Elov1* (fatty acid elongase1) and *Elov3* (fatty acid elongase3) involved in FA elongation. (**f**) *Fas* (fatty acid synthase), *Scd1* (stearoyl-coA desaturase 1) and *Scd2* (stearoyl-coA desaturase 2) involved in lipogenesis. Mean ± SEM; n = 3 for KO aged male and female, n = 4 for WT young and aged female and WT aged male, and n = 6 for young WT male and female and young KO male). In the graph, *p*-values are from Tukey’s post hoc tests between pairwise groups. Next to the graph, *p*-values for sex, age and SelenoN effect are defined in Table 2.

To determine whether a specific pathway could be responsible for male and female PL content differences in the diaphragm, we investigated gene expression involved in phospholipid biosynthesis and/or remodeling. Global sex effects were observed for mRNA expression of Fabp3, Sms1, Crsl1, AcadM, AcadVL, Hadha and Elov1, with higher gene expression levels in males than in females (cf. Figure 7a–d). These results could explain in part the multiple sex variations detected for PL diaphragm content.

Regarding aging, we noted an important decrease in gene expression of Hacd1, an enzyme involved in fatty acid elongation, with four times less transcript for aged females than for young ones and three times less for aged males compared to young ones, in both cases regardless of the genotype (cf. Figure 7e). Similarly, mRNA level of Elov1, encoding fatty acid elongase 1, was lower for aged animals than young ones but not to the same extend as for Hacd1. No significant change was observed for Elov3 (Figure 7e). Moreover, an increase in gene expression for Fas and the desaturases Scd1 and Scd2 was observed for aged animals compared to young animals, in both cases regardless of the sex and the genotype (cf. Figure 7f). Taken together, these results show an age-related decrease of elongase involved in carbon length and increase in desaturase catalysing the biosynthesis of monosatured fatty acid and a sex-specific remodeling of key enzymes required for fatty acid beta oxidation, transport, and for SM and CL biosynthesis.

## 4. Discussion

Lipids are an essential building block for cellular architecture and cell signaling [2,24]. Imbalances of phospholipids and sphingolipids, two important categories of lipids, can disrupt cell and organelle homeostasis and participate in various pathologies [4]. The indisputable evidence of the latter points comes from rare genetic diseases classified under the orphaned code ORPHA:352301 and which include mild to severe phenotypes involving the central nervous system, peripheral nerves or skeletal muscle, due to disorders of PL, sphingolipids and fatty acid synthesis.

In this study, we focused on lipid content in diaphragms from male and female, young (1 month) and aged (20 months), wild type and *SelenoN* KO mice. Using an unbiased approach, we found an important sex-specific phospholipid fingerprint through PL profiling in basal conditions as well as during aging and in selenopathy mice models. This finding is in congruence with several pathologies reported to exhibit sex specific characteristics such as cardiovascular diseases, diabetes, neurological disorders [25,26,27,28] as well as in muscular diseases [29]. Human longevity is also markedly influenced by sex [30]. However, the majority of the research studies have been done mainly in one sex. In 2010, Zucker and Beery pointed out that males still dominate animal studies [31]. In muscle, sex- and age-related changes have been described in term of fiber type composition, in metabolic capacities and in mitochondria content [18,29]. Nevertheless, there is limited information supporting the lipid variation mechanism due to sexual dimorphism. We have shown previously that PL composition presents some significant difference in male and female hearts in basal conditions that increase after doxorubicin treatment [23]. Recently, a sphingolipid mapping in 21 murine tissues revealed higher similarities than discrepancies for the 114 identified sphingolipids between males and females for most tissues [32]. Kidney, lung, skin, liver, and brown adipose tissue showed significant sex differences, but not in the skeletal muscle analyzed (the quadriceps muscle) [32]. The results, combined with our analysis, suggest that PL sexual dimorphism occurs in a muscle- and time-dependent manner.

PL composition can be influenced by diet. Two essential FAs cannot be synthetized in mammals, α-linolenic acid (C18:3, n-3) and linoleic acid (C18:2, n-6), and are found in food supply. Studies have shown that a high fat diet had an impact on phospholipid content and/or FA composition in muscle, which is not equivalent, depending on muscle fiber type [33], in liver [34], as well as in retina [35]. PL and sphingolipid content probably influence the overall structural architecture of the cells but also the organelles. Two of them are especially important for energy homeostasis and calcium-controlled-muscle contraction, namely mitochondria and the sarco-endoplasmic reticulum (SR/ER). Interestingly, the bulk of PL synthesis takes place in the endomembrane compartment, mainly the ER. Concerning mitochondria, the double membrane-bound organelle orchestrates the synthesis of PE and PC [36]. We show here that one of the most important sex differences is the decrease in content of PE and LPE specifically in males. This may be associated with the sexual dimorphism of mitochondria that has been observed in many pathologies with sex specificity [37]. Phospholipid trafficking occurs through the mitochondria-associated membranes (MAM), discovered six decades ago [38]. MAM are also considered as a specific calcium and reactive oxygen species interface [39]. While there are few phospholipidomic data for mitochondria, there are even fewer for SR/ER and MAM. While the SELENON protein is enriched in MAMs [19], we found the impact of SELENON deficiency in PL content was much less important than that of sex and age. Further research based on PL content of the organelles and MAM should help to clarify these points. Moreover, we showed an upregulation of selected genes expression involved in biosynthesis and remodeling of phospholipids (*Fabp3*, *Sms1*, *Crsl1*, *AcadM*, *AcadVL*, *Hadha* and *Elov1*) in the diaphragm of males compared to females. The sex-specific diaphragm fingerprint probably involves multiparametric and complex mechanisms.

Age-associated dysfunctions are involved in many features, including alteration of the local coupling between mitochondria and ER [40]. Lipid transfer, redox signaling and mitochondria dynamics have been shown to contribute to aging in numerous diseases [40]. We observed a decrease of the gene expression of *Hacd1* and an increase for two desaturases, *Scd1* and *Scd2*, in the older subjects compared to the younger. The desaturase allows the production of monounsaturated fatty acids, even if their level in the diet is already high [41]. We observed, from all the PL identified, that the composition of the fatty acyl chain is mainly of saturated or monounsaturated species. This composition certainly influences the fluidity of membranes, which will be less fluid than the ones containing unsaturated FA, leading to modifications of cell signaling [42]. Of note, mutations in *HACD1* gene encoding an enzyme involved in the modulation of very-long-chain fatty acids, have been associated with a congenital myopathy. The murine *Hacd1* KO model presents similarities with the *SelenoN* KO model; they both have reduction of muscle mass with metabolic abnormalities [19,43]. The absence of HACD1 leads to the decrease of CL content coupled with lower mitochondrial ATP production [43].

Cardiolipin content is an important parameter for energy production as optimal ATP production is linked to CL [44,45]. We identified 70 different cardiolipins; some are modulated by sex and SELENON absence and the most influencing parameter is age. Only cardiolipins composed of 70 carbon-length present increased oxidized CL during aging, whereas oxidized CL with 72 and 74 decreased. As oxidation is one of the theories for aging due to the damage produced by free radicals [46] and phospholipids being particularly vulnerable to oxidation, it is not surprising that lipid metabolism and signaling have an important role in aging and longevity [47]. Thus, a specific CL oxidation fingerprint was present in aged animals.

Cardiolipin and sphingomyelin remodeling was a hallmark of SELENON absence. Gene mutations in *SELENON* lead to a muscle disorder (SELENON(SEPN1)-Related Myopathy) with muscle weakness predominantly affecting neck and trunk muscles, and life-threatening respiratory insufficiency [16] largely due to diaphragmatic dysfunction [15]. As mentioned above, SELENON is enriched in the MAM and is involved in calcium and redox homeostasis [48]. In this study, we observed in the absence of SELENON 20 significant changes in PL composition in the diaphragm. This might contribute to the bioenergetics defect previously associated with SELENON-RM [19]. Interestingly, in muscle biopsies from patients affected by another inherited muscle disease, Duchenne muscular dystrophy, an unbiased ultra-high-resolution mass spectrometry imaging revealed significant changes in 34 metabolites, more than half of them being phospholipids [49]. Moreover, age is an important factor in the modulation of PL content in muscles from the murine model of Duchenne, the mdx mouse [50]. Our study expands these previous data and suggests that PL fingerprints may represent an important player in rare muscle diseases or in aging, with sex differences that need to be taken into account.

## 5. Conclusions

Phospholipids are a vast family with various structures that present specific properties. Their role in muscle physiology and disease has been under-investigated. Using mass spectrometry, we identified 191 phospholipids in the diaphragm. More than half of them are significantly different between males and females, 40% are modulated by age and 10% by the absence of SELENON, which represents a pathophysiological model of congenital myopathies. Integrating sex analysis is an essential step of personalized medicine, avoiding the pitfall of taking half of the human population as representative of the whole and potentially revealing opportunities for novel discoveries. Along these lines, skeletal muscle, the largest and one of the most metabolically active tissues in the human body, shows sex differences, including PL composition, that should not be ignored.

## Figures and Tables

**Table 1:** Sequence of qPCR primers.

Gene	Forward Primer	Reverse Primer
*Rpl19* *Hprt* *Fabp3* *Sms1* *Crls1* *AcadM* *AcadVL* *Hadha* *Elov1* *Elov3* *Fas* *Scd1* *Scd2*	GGGCAGGCATATGGGCATA AGCTACTGTAATGATCAGTCAACG TTCTGGAAGCTAGTGGACAG TCCATCACAGGCTCGCACA TGACCTATGCAGATCTTATTCCA CCGTTCCCTCTCATCAAAAG TGGGCCTCTCTAATACCCAGT GCTTGGGAGGAGGACTTGA GGCCCTGATCCCTTTGAA GCACCATCTTTGGCATACTG GCAAGTGCAGCCTGAGGGAC CTACATGACCAGCGCTCTG GTAATCAGCGCCCTGGGCAT	GGCGGTCAATCTTCTTGGATT AGAGGTCCTTTTCACCAGCA TGATGGTAGTAGGCTTGGTCAT ACGCTGAGGAGCCAGCAAA CCTTGCTGATGAATGTTGGTT ACACCCATACGCCAACTCTT TCCCAGGGTAACGCTAACAC AGCAACACTTCAGGGACACC CCATCCTGGCTAAGGACTCA TTGTGGGTGTGGCATCCTTTC ACAGCCTGGGGTCATCTTTGC CGTACACGTCATTCTGGAACG CATACACGTCATTCTGGAACGC

**Table 2:** Sex-significant PLs in diaphragm.

Detailed Identification	Global Identification	*m*/*z*	p_SEX	p_AGE	p_SELENON
ND	CL(66:4)	1371.90	0.0168	0.0016	0.0026
ND	CL(66:5)	1369.91	0.0148	0.74	0.18
16:0-16:1-18:1-18:2 + 14:0-(18:1)2-18:2	CL(68:4)	1399.97	0.0027	0.0035	0.0403
14:0-18:1-(18:2)2 + (16:1)2-18:1-18:2	CL(68:5)	1397.95	0.0025	0.053	0.08
ND	CL(68:6)	1395.93	0.0026	0.07	0.62
16:0-(18:1)3	CL(70:3)	1430.00	0.054	0.06	0.0199
16:0-(18:1)2-18:2	CL(70:4)	1427.99	0.0369	0.0259	0.0136
16:1-(18:1)2-18:2 + 16:0-18:1-(18:2)2	CL(70:5)	1425.98	0.0008	0.0016	0.08
16:1-(18:2)2-18:1 + 16:0-(18:2)3	CL(70:6)	1423.97	0.0003	0.07	0.51
16:1-(18:2)3	CL(70:7)	1421.95	0.0003	0.86	0.70
	CL(70:7. +1O)	1437.95	0.0029	0.053	0.89
	CL(70:7. +3O)	1469.94	0.0020	0.0289	0.33
(18:1)3-18:2 + 18:0-18:1-(18:2)2	CL(72:5)	1454.00	0.0110	0.0374	0.11
(18:1)2-(18:2)2	CL(72:6)	1451.99	0.0065	0.25	0.31
	CL(72:6. +3O)	1499.99	0.0089	0.26	0.20
18:1-(18:2)3	CL(72:7)	1449.98	0.0029	0.0072	0.69
	CL(72:7. +3O)	1497.97	0.0044	0.71	0.32
	CL(72:7. +6O)	1545.95	0.0411	0.32	0.76
(18:2)4	CL(72:8)	1447.96	0.0018	0.0008	0.78
	CL(72:8. +3O)	1495.95	0.0014	0.15	0.81
	CL(72:8. +5O)	1527.94	0.0447	0.09	0.85
	CL(72:8. +6O)	1543.93	0.0151	0.86	0.68
	CL(72:8. +9O)	1591.93	0.0182	0.12	0.94
(18:2)3-18:3	CL(72:9)	1445.95	0.0039	0.29	0.06
ND	CL(74:10)	1471.96	0.0046	0.27	0.26
18:1-(18:2)2-20:2 + (18:1)2-18:2-20:3 + 18:0-18:1-18:2-20:4	CL(74:7)	1478.01	0.0475	0.000002	0.10
(18:2)3-20:2 + 18:1-(18:2)2-20:3	CL(74:8)	1475.99	0.0066	0.000002	0.37
	CL(74:8. +3O)	1523.99	0.0132	0.0006	0.26
(18:2)3-20:3 + 18:1-(18:2)2-20:4	CL(74:9)	1473.98	0.0086	0.06	0.78
	CL(74:9. +3O)	1521.97	0.0047	0.0446	0.64
ND	CL(76:8)	1504.02	0.0143	0.00002	0.17
ND	CL(76:9)	1502.00	0.0249	0.16	0.27
16:0	LPE(16:0)	452.28	0.0007	0.0112	0.14
18:0	LPE(18:0)	480.31	0.00003	0.0097	0.07
18:1	LPE(18:1)	478.29	0.0090	0.21	0.08
18:2	LPE(18:2)	476.28	0.0043	0.0232	0.42
20:4	LPE(20:4)	500.28	0.0035	0.60	0.12
22:6	LPE(22/6)	524.28	0.0010	0.34	0.60
22:5	LPE(22:5)	526.29	0.0097	0.0523	0.30
16:0-14:0	PC(30:0)	764.54	0.0236	0.0035	0.09
18:1-18:0 + 20:1-16:0	PC(36:1)	846.62	0.0002	0.07	0.0389
18:0-18:1	PC(36:1)	772.58	0.00003	0.0401	0.0401
18:2-18:0 + 18:1-18:1 + 16:0-20:2	PC(36:2)	844.61	0.0092	0.0006	0.50
16:0-20:4 + 18:2-18:2	PC(36:4)	840.57	0.0013	0.0081	0.80
20:4-18:0 + 22:4-16:0	PC(38:4)	868.60	0.00002	0.0130	0.34
22:5-16:0 + 20:4-18:1	PC(38:5)	866.59	0.0103	0.16	0.37
22:6-16:0	PC(38:6)	864.57	0.0040	0.87	0.69
ND	PC(39:3(OH))	876.59	0.0272	0.31	0.46
ND	PC(39:4(OH))	874.58	0.0100	0.52	0.33
18:0-22:5	PC(40:5)	894.61	0.0017	0.52	0.12
18:0-22:6	PC(40:6)	892.61	0.0005	0.53	0.36
22:6-18:1 + 22:5-18:2 + 20:3-20:4	PC(40:7)	890.59	0.0256	0.89	0.59
ND	PC(44:8)	870.59	0.0000	0.11	0.18
ND	PC(46:11(OH))	908.58	0.0210	0.21	0.14
ND	PE(32:0)	690.51	0.0232	0.51	0.21
16:0-18:1 + 16:1-18:0	PE(34:1)	716.52	0.0013	0.77	0.10
16:0-18:2 + 16:1-18:1	PE(34:2)	714.51	0.0387	0.0063	0.0494
18:0-18:1	PE(36:1)	744.55	0.0226	0.0233	0.0320
18:0-18:2 + 18:1-18:1	PE(36:2)	742.54	0.0008	0.0092	0.46
20:3-16:0 + 18:2-18:1	PE(36:3)	740.52	0.0065	0.0207	0.59
20:4-16:0 + 18:2-18:2	PE(36:4)	738.51	0.0009	0.0395	0.54
ND	PE(38:4(OH)) + PE(P-36:4)	782.53	0.0058	0.0036	0.33
20:4-18:0	PE(38:4)	766.54	0.0003	0.0033	0.62
22:5-16:0	PE(38:5)	764.52	0.0021	0.0462	0.31
ND	PE(38:6(OH))	778.50	0.0009	0.54	0.51
ND	PE(38:6(OH)) + PE (41:7)	838.52	0.0041	0.17	0.28
22:6-16:0	PE(38:6)	762.51	0.0003	0.76	0.32
22:5-18:0 + 22:4-18:1	PE(40:5)	792.55	0.0007	0.48	0.24
ND	PE(40:6(OH))	806.53	0.0002	0.22	0.19
22:6-18:0 + 22:5-18:1	PE(40:6)	790.54	0.0002	0.79	0.40
22:6-18:1 + 22:5-18:2	PE(40:7)	788.52	0.0187	0.20	0.89
ND	PE(O-32:1) + PE(P-32:0)	674.51	0.0005	0.0244	0.68
ND	PE(O-34:1) + PE(P-34:0)	702.54	0.0011	0.86	0.35
PE(P-16:0-18:1)	PE(O-34:2) + PE(P-34:1)	700.53	0.0034	0.99	0.47
ND	PE(O-34:3) + PE(P-34:2)	698.51	0.0010	0.0166	0.73
PE(P-18:0-18:1)	PE(O-36:2) + PE(P-36:1)	728.56	0.0268	0.0122	0.38
PE(P-18:1-18:1)	PE(O-36:3) + PE(P-36:2)	726.54	0.0295	0.0160	0.51
PE(P-16:0-20:4)	PE(O-36:5) + PE(P-36:4)	722.51	0.0009	0.20	0.15
ND	PE(O-38:4) + PE(P-38:3)	752.56	0.0011	0.52	0.13
PE(P-18:0-20:4) + PE(P-16:0-22:4)	PE(O-38:5) + PE(P-38:4)	750.54	0.0007	0.28	0.19
PE(P-18:0-22:5)	PE(P-18:0-22:5)	776.56	0.0010	0.68	0.27
ND	PE(P-32:1)	672.50	0.0022	0.14	0.35
PE(P-16:0-22:6)	PE(P-38:6)	746.51	0.0004	0.91	0.77
PE(P-18:0-22:6)	PE(P-40:6)	774.54	0.0002	0.21	0.47
(16:0)2	PI(32:0)	809.52	0.0048	0.0040	0.0212
ND	PI(32:2)	865.51	0.0007	0.18	0.97
ND	PI(34:3)	891.52	0.0021	0.62	0.89
ND	PI(36:3(OH))	875.53	0.0013	0.39	0.19
20:4-16:0	PI(36:4)	857.52	0.0011	0.0358	0.14
ND	PI(36:5)	915.52	0.0313	0.15	0.90
18:0-20:3	PI(38:3)	887.56	0.0012	0.68	0.60
18:0-20:4	PI(38:4)	885.55	0.0002	0.52	0.46
18:1-20:4 + 18:0-20:5 + 16:0-22:5	PI(38:5)	883.53	0.0031	0.32	0.24
ND	PI(38:6)	881.52	0.0013	0.07	0.18
18:0-22:4	PI(40:4)	913.57	0.0180	0.0069	0.13
18:0-22:5	PI(40:5)	911.56	0.0292	0.0115	0.18
18:0-22:6	PI(40:6)	909.55	0.0031	0.53	0.24
ND	PI(P-37:2)	895.54	0.0005	0.48	0.68
18:1-18:0	PS(36:1)	788.54	0.0403	0.0450	0.0453
20:3-18:0	PS(38:3)	812.54	0.0013	0.12	0.07
18:0-20:4 + 18:1-20:3	PS(38:4)	810.53	0.0001	0.59	0.13
22:6-18:0	PS(40:6)	834.53	0.0001	0.70	0.56
d18:1/16:0	SM(d34:1)	761.58	0.0344	0.71	0.00
d18:1/18:0	SM(d36:1)	765.57	0.0143	0.40	0.43
d18:1/18:1	SM(d36:2)	787.60	0.0021	0.0003	0.11
d18:1/22:0 + d18:1/24:2	SM(d40:1) + SM(d42:3)	845.67	0.0315	0.0338	0.10
d18:1/23:0	SM(d41:1)	859.69	0.0278	0.0023	0.48
ND	SM(d41:2)	857.67	0.0231	0.0195	0.64
d18:1/24:0	SM(d42:1)	873.70	0.0388	0.08	0.0234
d18:1/24:1	SM(d42:2)	871.69	0.0464	0.17	0.0147

ND Not determined. Three-way analysis of variance including AGE, GENOTYPE and SEX factor with interactions was performed. p_SEX is for the comparison of all males versus all females (whatever the age and the genotype). p_AGE is for the comparison of all young versus all aged animals (whatever the sex and the genotype). p_SELENON is for the comparison of all WT versus all KO animals (whatever the sex and the age). Red color corresponds to a *p*-value < 0.05.

**Table 3:** Age-significant PL in diaphragm.

Detailed Identification	Global Identification	*m*/*z*	p_SEX	p_AGE	p_SELENON
ND	CL(66:4)	1371.90	0.0168	0.0016	0.0026
16:0-16:1-18:1-18:2 + 14:0-(18:1)2-18:2	CL(68:4)	1399.97	0.0027	0.0035	0.0403
14:0-18:1-(18:2)2 + (16:1)2-18:1-18:2	CL(68:5)	1397.95	0.0025	0.0529	0.08
16:0-(18:1)2-18:2	CL(70:4)	1427.99	0.0369	0.0259	0.0136
16:1-(18:1)2-18:2 + 16:0-18:1-(18:2)2	CL(70:5)	1425.98	0.0008	0.0016	0.08
	CL(70:5, +2O)	1457.97	0.09	0.0009	0.80
	CL(70:6, +2O)	1455.97	0.30	0.0001	0.46
	CL(70:6, +4O)	1487.95	0.90	0.0338	0.49
	CL(70:7, +1O)	1437.95	0.0029	0.0531	0.89
	CL(70:7, +3O)	1469.94	0.0020	0.0289	0.33
(18:1)3-18:2 + 18:0-18:1-(18:2)2	CL(72:5)	1454.00	0.0110	0.0374	0.11
18:1-(18:2)3	CL(72:7)	1449.98	0.0029	0.0072	0.69
	CL(72:7, +1O)	1465.97	0.54	0.0527	0.29
(18:2)4	CL(72:8)	1447.96	0.0018	0.0008	0.78
	CL(72:8, +1O)	1463.96	0.19	0.0029	0.87
	CL(72:8, +2O)	1479.96	0.31	0.0009	0.38
	CL(72:9, +1O)	1461.94	0.09	0.0031	0.98
	CL(72:9, +3O)	1493.93	0.09	0.0220	0.98
	CL(72:9, +5O)	1525.94	0.10	0.0305	0.46
18:1-(18:2)2-20:2 + (18:1)2-18:2-20:3 + 18:0-18:1-18:2-20:4	CL(74:7)	1478.01	0.0475	0.000002	0.10
ND	CL(74:7, +2O)	1510.00	0.17	0.00002	0.73
ND	CL(74:7, +4O)	1541.98	0.35	0.0015	0.89
ND	CL(74:7, +5O)	1557.95	0.43	0.0019	0.72
(18:2)3-20:2 + 18:1-(18:2)2-20:3	CL(74:8)	1475.99	0.0066	0.000002	0.37
ND	CL(74:8, +1O)	1491.99	0.08	0.0000003	0.28
ND	CL(74:8, +2O)	1507.98	0.66	0.0000002	0.57
ND	CL(74:8, +3O)	1523.99	0.0132	0.0006	0.26
ND	CL(74:8, +4O)	1539.97	0.56	0.00001	0.95
ND	CL(74:8, +5O)	1555.97	0.96	0.000001	0.19
ND	CL(74:8, +6O)	1571.97	0.10	0.0001	0.82
ND	CL(74:9, +1O)	1489.97	0.06	0.0128	0.87
ND	CL(74:9, +2O)	1505.97	0.14	0.0001	0.72
ND	CL(74:9, +3O)	1521.97	0.0047	0.0446	0.64
ND	CL(74:9, +4O)	1537.96	0.97	0.0047	0.47
ND	CL(76:8)	1504.02	0.0143	0.00002	0.17
16:0	LPE(16:0)	452.28	0.0007	0.0112	0.14
18:0	LPE(18:0)	480.31	0.00003	0.0097	0.07
18:2	LPE(18:2)	476.28	0.0043	0.0232	0.42
22:5	LPE(22:5)	526.29	0.0097	0.052	0.30
16:0-14:0	PC(30:0)	764.54	0.0236	0.0035	0.08
16:0-16:0	PC(32:0)	768.53	0.29	0.0010	0.19
18:2-16:0 + 18:1-16:1	PC(34:2)	816.57	0.98	0.00002	0.47
ND	PC(34:3)	814.56	0.94	0.0069	0.99
18:0-18:1	PC(36:1)	772.58	0.00001	0.0401	0.0401
18:2-18:0 + 18:1-18:1 + 16:0-20:2	PC(36:2)	844.61	0.0092	0.0006	0.50
18:1-18:2 + 16:0-20:3	PC(36:3)	842.59	0.43	0.0137	0.41
16:0-20:4 + 18:2-18:2	PC(36:4)	840.57	0.0013	0.0081	0.80
20:4-18:0 + 22:4-16:0	PC(38:4)	868.60	0.0000	0.0130	0.34
16:0-18:2 + 16:1-18:1	PE(34:2)	714.51	0.0387	0.0063	0.0494
18:0-18:1	PE(36:1)	744.55	0.0226	0.0233	0.0320
18:0-18:2 + 18:1-18:1	PE(36:2)	742.54	0.0008	0.0092	0.46
20:3-16:0 + 18:2-18:1	PE(36:3)	740.52	0.0065	0.0207	0.59
20:4-16:0 + 18:2-18:2	PE(36:4)	738.51	0.0009	0.0395	0.54
ND	PE(38:4(OH)) + PE(P-36:4)	782.53	0.0058	0.0036	0.33
20:4-18:0	PE(38:4)	766.54	0.0003	0.0033	0.62
22:5-16:0	PE(38:5)	764.52	0.0021	0.0462	0.31
ND	PE(O-32:1) + PE(P-32:0)	674.51	0.0005	0.0244	0.68
ND	PE(O-34:3) + PE(P-34:2)	698.51	0.0010	0.0166	0.73
PE(P-18:0-18:1)	PE(O-36:2) + PE(P-36:1)	728.56	0.0268	0.0122	0.38
PE(P-18:1-18:1)	PE(O-36:3) + PE(P-36:2)	726.54	0.0295	0.0160	0.51
18:2-16:0 + 18:1-16:1	PG(34:2)	745.50	0.06	0.0514	0.19
16:0-19:1 + 17:0-18:1	PG(35:1)	761.53	0.29	0.0453	0.09
(16:0)2	PI(32:0)	809.52	0.0048	0.0040	0.0212
18:2-16:0 + 18:1-16:1 + 18:0-16:2	PI(34:2)	833.52	0.47	0.0060	0.36
18:0-18:1	PI(36:1)	863.56	0.97	0.0139	0.0422
20:4-16:0	PI(36:4)	857.52	0.0011	0.0358	0.14
ND	PI(38:6)	941.54	0.17	0.0351	0.42
18:0-22:4	PI(40:4)	913.57	0.0180	0.0069	0.13
18:0-22:5	PI(40:5)	911.56	0.0292	0.0115	0.18
18:1-18:0	PS(36:1)	788.54	0.0403	0.0450	0.0453
d18:1/18:1	SM(d36:2)	787.60	0.0021	0.0003	0.11
d18:1/22:0 + d18:1-24:2	SM(d40:1) + SM(d42:3)	845.67	0.0315	0.0338	0.10
d18:1/23:0	SM(d41:1)	859.69	0.0278	0.0023	0.48
ND	SM(d41:2)	857.67	0.0231	0.0195	0.64
d18:1/24:2 + d18:2/24:1	SM(d42:3)	869.67	0.055	0.00003	0.0094

Red color corresponds to a *p*-value < 0.05.

**Table 4:** *SelenoN* (genotype) significant PL in diaphragm.

Detailed Identification	Global Identification	*m*/*z*	p_SEX	p_AGE	p_SELENON
ND	CL(66:4)	1371.90	0.0168	0.0016	0.0026
ND	CL(68:1)	1406.00	0.10	0.20	0.0047
(16:0)2-(18:1)2	CL(68:2)	1404.00	0.34	0.51	0.0031
16:0-16:1-(18:2)2 + (16:0)2-18:1-18:2	CL(68:3)	1401.98	0.13	0.94	0.0039
16:0-16:1-18:1-18:2 + 14:0-(18:1)2-18:2	CL(68:4)	1399.97	0.0027	0.0035	0.0403
16:0-(18:1)3	CL(70:3)	1430.00	0.054	0.058	0.0199
16:0-(18:1)2-18:2	CL(70:4)	1427.99	0.0369	0.0259	0.0136
18:0-18:1	PC(36:1)	772.58	0.00003	0.0401	0.0401
16:0-18:2 + 16:1-18:1	PE(34:2)	714.51	0.0387	0.0063	0.0494
18:0-18:1	PE(36:1)	744.55	0.0226	0.0233	0.0320
14:0-16:0	PG(30:0)	693.47	0.08	0.70	0.0156
(16:0)2	PI(32:0)	809.52	0.0048	0.0040	0.0212
18:0-18:1	PI(36:1)	863.56	0.97	0.0139	0.0422
18:1-18:0	PS(36:1)	788.54	0.0403	0.0450	0.0453
18:1-18:1 + 18:0-18:2	PS(36:2)	786.53	0.08	0.47	0.0232
d18:1/16:0	SM(d34:1)	761.58	0.0344	0.71	0.0006
ND	SM(d34:2)	759.57	0.16	0.07	0.0034
d18:1/24:0	SM(d42:1)	873.70	0.0388	0.08	0.0234
d18:1/24:1	SM(d42:2)	871.69	0.0464	0.17	0.0147
d18:1/24:2 + d18:2/24:1	SM(d42:3)	869.67	0.055	0.00003	0.0094

Red color corresponds to a *p*-value <0.05.

## Data Availability

All data are available upon request.
